# Use of MRI for Risk Stratification in Anticoagulation Decision Making in Atrial Fibrillation: Promising, but More Data are Needed for a Robust Algorithm

**DOI:** 10.3389/fneur.2014.00003

**Published:** 2014-01-20

**Authors:** Duncan Wilson, Andreas Charidimou, David J. Werring

**Affiliations:** ^1^Stroke Research Group, National Hospital for Neurology and Neurosurgery, UCL Institute of Neurology, London, UK

**Keywords:** cerebral microbleeds, intracerebral hemorrhage, risk stratification, amyloid angiopathy, MRI imaging

We read with interest Mark Fisher’s review paper highlighting the very difficult decision-making many stroke physicians and neurologists are facing around the world on chronic anticoagulation for atrial fibrillation (AF) (1). As the author points out, oral anticoagulants are underutilized, often based on erroneous clinical reasoning, which may over-estimate bleeding risks. Part of the problem is that current clinical scoring systems for bleeding risk (e.g., HEMORR2HAGES, ATRIA, and HAS-BLED) might be of limited value in everyday clinical practice, especially in regard to intracerebral hemorrhage (ICH), the most feared and devastating complication of anticoagulation (2). The development of advanced brain MR imaging provides unique promise to tailor individual treatment decisions on anticoagulation by better balancing ICH and ischemic stroke risks (2). New radiological markers of cerebral small vessel disease (including cerebral microbleeds, cortical superficial siderosis, and white matter changes, etc.) have the potential to provide information about the presence of a hemorrhage-prone microangiopathy, which seems to underlie anticoagulation-related ICH (3–5).

We applaud the author’s new algorithm incorporating cerebral microbleeds on blood-sensitive MRI sequences (1); however, before this approach can be recommended in clinical practice some potential limitations should be considered. First, the data used to support the new algorithm come from a heterogeneous group of AF or stroke patients from very different study designs. In some of these studies patients had suffered spontaneous ICH (6, 7), in others previous ischemic stroke (7–9), or no previous event (7). Second, evidence largely comes from case–control studies, which cannot prove causality. Third, data from patients with different ethnic backgrounds might not be generalizable to all populations (6, 8). For example, the largest prospective study on CMBs and stroke risk after ischemic stroke to date included an Eastern (Asian) population, and the vast majority (93.4%) of patients with subsequent ICH had deep CMBs likely reflecting the high prevalence of hypertensive arteriopathy, with a low prevalence of cerebral amyloid angiopathy in this cohort. The distribution of CMBs may be relevant for risk models. Furthermore, in a recent meta-analysis of CMBs in ischemic stroke patients (9) the risk of ICH increased up to eightfold in those with CMB vs. those without, while the overall stroke risk seemed to double. However, the association between CMBs and subsequent ICH was much stronger for Eastern (Asian) compared to Western populations. These data suggest that indeed in a subgroup of patients, CMBs can potentially tip the balance away from net clinical benefit for anticoagulation, but this may not be generalizable across populations of different ancestry. It must also be noted that in some populations CMBs also confer a risk of future ischemic stroke as well as ICH, with no studies clearly addressing the balance of future cerebral bleeding vs. ischaemia (10).

A new algorithm that incorporates MRI to tailor individual treatment decisions on anticoagulation in AF patients could also take into account other hemorrhagic and ischemic markers of cerebrovascular disease, such as cortical superficial siderosis, small ischemic lesions (acute or chronic), and white matter changes. Finally, any algorithm that looks to tailor individual treatment decisions in AF should also incorporate alternative non-pharmacological treatments. This is most pertinent in those cases where anticoagulation is contraindicated, or when the risks of warfarin outweigh the benefits.

The vast majority of thrombus formation in AF occurs in the left atrial appendage. There is good evidence that closure of this appendage can reduce risk of ischaemic stroke (11–14), with demonstration of non-inferiority to warfarin (15). All left atrial appendage devices however are still under evaluation, with current evidence based on limited follow up periods, small study numbers, and carry risks of surgical complications. Moreover, both the WATCHMAN and Amplatzer devices require a short period of dual antiplatelet therapy followed by life-long monotherapy. It is for these reasons that the European Society of Cardiology guidelines for the management of AF gave these devices a grade 2B recommendation, which is: “consider in patients with thromboembolic risk who cannot be managed in the long-term using any form of OAC” (16).

The innovative paper by Fisher et al. is an important first step in personalizing anticoagulation treatment for AF. However, larger prospective studies using standardized MRI in a range of populations treated with anticoagulants for AF patients are urgently needed to provide reliable data to include in new treatment algorithms (for example http://www.ucl.ac.uk/cromis-2) (17). Such algorithms will then need to be validated in other large populations before they can truly inform clinical practice.
